# Flexor Tendon Repair Using a New Looped Six- and Eight-Strand Technique—A Biomechanical Analysis

**DOI:** 10.3390/jpm16030144

**Published:** 2026-03-03

**Authors:** Lucas G. de Groot, Caroline A. Hundepool, Jaimy E. Koopman, Pierluigi Tos, Jelle M. Zuidam

**Affiliations:** 1 Department of Plastic, Reconstructive and Hand Surgery, Erasmus MC University Medical Center, 3015 GD Rotterdam, The Netherlands; l.g.degroot@erasmusmc.nl (L.G.d.G.);; 2 Hand Surgery and Reconstructive Microsurgery Department, ASST Gaetano Pini-CTO, 20122 Milan, Italy

**Keywords:** tendon injuries, flexor tendon repair, suture techniques, biomechanical analysis, personalized medicine

## Abstract

**Background/Objectives:** Tendon injuries are a common cause of emergency department presentation and impose a substantial socioeconomic burden. Despite advances in surgical techniques, rupture rates after primary repair remain at 3.1–11.7%. Contemporary repairs typically combine at least four core strands with epitenon sutures to achieve sufficient tensile strength while limiting bulk. Increasing the number of core strands improves strength but may impair gliding and healing. Looped core sutures increase the effective strand number without additional knots or passes, potentially allowing omission of the epitenon suture and thus limiting repair complexity and bulk. The objective was to determine whether six- or eight-strand looped core suture techniques provide sufficient tensile strength to allow omission of an epitenon suture without excessive repair bulk, compared with a conventional four-strand Adelaide repair. **Methods:** One hundred and twenty human flexor digitorum profundus tendons were harvested from fresh-frozen anatomical specimens and allocated to six groups: Adelaide (four-strand) ± epitenon suture, six-strand ± epitenon suture, and eight-strand ± epitenon suture. Repairs were performed in zone II. The cross-sectional area (CSA) was measured before and after repair to quantify bulkiness. Tendons were tested to failure using axial tensile loading, and the failure mode was recorded. **Results:** The Adelaide with epitenon suture, six-strand with epitenon suture, and eight-strand with epitenon suture demonstrated significantly higher load to failure than the Adelaide without epitenon suture. The eight-strand without epitenon suture achieved a load to failure comparable to the Adelaide with epitenon suture, while also resulting in a smaller increase in CSA. The Adelaide with epitenon suture showed the greatest increase in CSA, while the six-strand without epitenon suture showed the smallest increase in CSA. Suture breakage was the predominant failure mode. **Conclusions:** An eight-strand looped core suture without epitenon suture provides comparable tensile strength to the conventional Adelaide repair with epitenon suture while minimizing repair bulk. The six-strand with epitenon suture demonstrated similar tensile strength to higher-strand techniques and may represent a mechanically adequate alternative with less tissue manipulation. These findings support a more individualized approach to flexor tendon repair, in which the choice of repair construct can be tailored to biomechanical demands and clinical context rather than applying a single uniform technique.

## 1. Introduction

Hand injuries are common amongst emergency department visits, with an estimated incidence of 896 hand and wrist injuries per 100,000 person years [1], of which 7 to 33.2 are estimated to involve flexor tendon injuries [2,3,4]. These injuries predominantly affect young, working-age men [2,3,4], resulting in a substantial socioeconomic burden due to lost productivity [5].

Primary repair of flexor tendon injuries remains challenging. Outcomes vary across different centers, with rupture rates following surgery ranging from 3.1 to 11.7% [4,6,7]. This variability may be attributed to differences in surgical technique, suture material, and rehabilitation protocol preferences between surgeons. Currently, the consensus includes either a four- or six-strand repair, with sparse epitenon sutures combined with an early active rehabilitation protocol [8]. However, for early active rehabilitation to be successful, the repair must withstand sufficient tensile forces to prevent rupture [8]. From a personalized medicine perspective, flexor tendon repair strategies may need to be adapted to patient- and injury-specific factors, including tendon quality, pulley integrity, anticipated rehabilitation demands, and surgeon experience [8].

One approach to achieving sufficient tensile strength in tendon repair is to increase the number of core sutures. Previous studies have shown that an eight-strand repair can withstand significantly higher loads than two- or four-strand repairs [9]. However, additional core strands add complexity and may also increase bulkiness at the repair site, which possibly impairs tendon gliding through the tendon sheath. Therefore, optimizing the balance between the number of core strands, complexity, and minimal bulkiness is essential.

Looped sutures make it possible to increase the number of core strands crossing the repair site without additional knots or extra suture passes, potentially enhancing strength while keeping the repair straightforward and low-profile. In this study, we compared looped six- and eight-strand core repairs with a conventional four-strand Adelaide technique, each performed with and without an epitenon suture. We evaluated ultimate load to failure and the change in cross-sectional area (CSA) to determine whether increasing the number of core strands can enhance repair strength while limiting repair bulkiness.

## 2. Materials and Methods

For this study, 120 flexor digitorum profundus (FDP) tendons were divided into six groups, with 20 tendons in each group. The tendons were allocated so that each group consisted of 5 tendons of the second through to the fifth digit. The suture techniques applied in each group were as follows:

Group I: 4-strand cruciate (Adelaide) without epitenon suture
Group II: 4-strand cruciate (Adelaide) with epitenon suture
Group III: 6-strand double loop without epitenon suture
Group IV: 6-strand double loop with epitenon suture
Group V: 8-strand double loop cruciate without epitenon suture
Group VI: 8-strand double loop cruciate with epitenon suture

**Figure 1:**
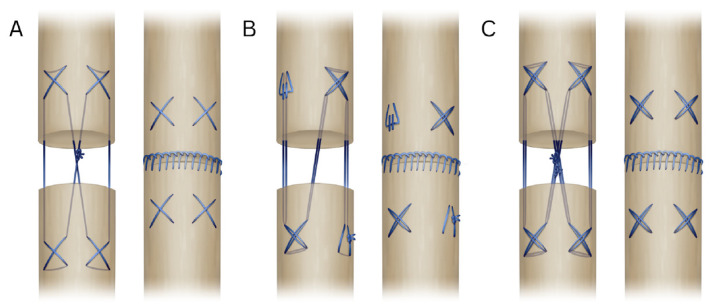
Schematic illustration showing the different suture techniques. (**A**). Four-strand cruciate (Adelaide) ± epitenon suture; (**B**). Six-strand double loop ± epitenon suture; (**C**). Eight-strand double loop ± epitenon suture.

The 4-strand cruciate (Adelaide) technique consists of four crosses, two on each side, followed by a knot in the middle, resulting in four strands crossing the core of the tendon. The 6-strand double loop starts by securing the suture through the end of the loop, followed by two crosses diagonally from each other, ending in tying the ends together diagonally from the start. This is a modified version of the 6-strand Lim/Tsai technique [10], in which the Lim/Tsai loops were replaced with cruciate crosses. The 8-strand double loop cruciate follows the same pattern as the Adelaide, but uses a looped suture, resulting in eight strands through the core of the tendon and two knots in the middle. For the epitenon suture, a running locking pattern was used (Figure 1).

Core sutures in the Adelaide ± epitenon suture groups were performed with Ethilon 4.0 sutures (Ethicon, Johnson & Johnson, Amersfoort, The Netherlands), while core sutures in the 6- and 8-strand ± epitenon suture groups were performed with 4.0 double-loop polyfilament caprolactam sutures (Supramid, S Jackson, Alexandria, VA, USA). The epitenon sutures were performed using Ethilon 5.0 sutures (Ethicon, Johnson & Johnson, Amersfoort, The Netherlands). All tendon reconstructions were performed by a single attending plastic surgeon who specialized in hand surgery.

A total of 120 FDP tendons were harvested from donated human upper limbs. Anatomical donations were obtained with informed consent for use in medical research. The limbs were fresh-frozen and thawed at room temperature on the day of tendon harvesting.

Tendons from the second to fifth digit were transversely cut in situ in zone II [11], after which they were sutured according to their assigned group. After suturing, the tendon was removed from the limb by transversely transecting the proximal and distal ends. Proximally, the tendon was transected distal to the musculotendinous junction, while the distal end was transected proximal to the FDP’s insertion on the distal phalanx. This method ensured that there was sufficient tendon length on both sides of the suture site for insertion in the testing machine clamps. After harvesting, tendon height and width were measured with digital calipers at the thickest point of the sutured tendon and at a location 5–10 mm proximal to the most proximal part of the suture, representing native tendon dimensions. After completion of all CSA measurements, the tendons were briefly (<2 min) wrapped in saline-soaked gauze during transfer to the testing machine to prevent dehydration.

Biomechanical testing was performed using an axial tension machine (Testometric M250-AX Materials Testing Machine, Testometric Co., Ltd., Rochdale, UK). First, the saline-soaked gauze, which was used to wrap the entire tendon to prevent dehydration, was removed. Then, the proximal and distal ends of the sutured tendon were wrapped in fresh saline-soaked gauze and inserted into serrated clamps to prevent slippage. Care was taken to center the suture site between the clamps and to ensure proper alignment to prevent off-axis loading. Tendons were pre-loaded with 2–5 Newton (N) to remove slack before testing. Testing was performed at room temperature with a distraction rate of 15 mm/minute until failure occurred. Failure was defined as suture breakage, slipping of the suture out of the tendon, or slipping of the tendon out of the clamp.

The height and width measurements of the tendon were used to calculate the CSA of the tendon at each site using the formula:

$$CSA\;=\;\pi \;\times \;(\frac{height}{2})\times (\frac{width}{2})$$

The percentage increase in CSA due to the suture repair was calculated as:

$$\%\;increase\;CSA=(\frac{CSA\;at\;suture\;site-CSA\;proximal\;to\;suture\;site}{CSA\;proximal\;to\;suture\;site})\times 100$$

These data were analyzed to quantify the effects of suture repair on tendon bulk. Load to failure was defined as the maximum force (N) recorded before the suture repair failed. Small gapping (<5 mm) at the suture site was visually assessed and deemed acceptable without being defined as failure. The load-to-failure data were used to calculate a mean and standard deviation (SD) for each group.

Statistical analysis was performed using a one-way ANOVA to compare load to failure and % increase in CSA across groups. Tukey’s post hoc corrections were applied to correct for multiple comparisons. Normal distribution of data and homogeneity of variance were determined using the Shapiro–Wilk test and Levene’s test, respectively. A chi-square test of independence was performed to examine whether the causes of failure differed between groups. When the overall test was significant, adjusted standardized residuals were examined to identify which group–cause combinations contributed most to the association. A *p*-value < 0.05 was deemed statistically significant. Results are presented as mean and SD. All statistical tests were performed using IBM SPSS Statistics (Version 28.0.1.0).

## 3. Results

For the load to failure and cause of failure analyses, all tendons with complete load to failure data were included, resulting in 20 tendons in the Adelaide ± epitenon group, six-strand with epitenon group, and eight-strand ± epitenon group. The six-strand without epitenon group had 19 tendons (Table 1). Of the 120 tendons used, CSA data were missing for 13 tendons due to digital caliper malfunction. These tendons were excluded from the CSA increase analysis, resulting in 16 tendons in the Adelaide ± epitenon and eight-strand without epitenon groups, 19 tendons in the six-strand without epitenon group, and 20 tendons in the six-strand with epitenon and eight-strand with epitenon groups (Table 2).

### 3.1. Load to Failure

**Figure 2:**
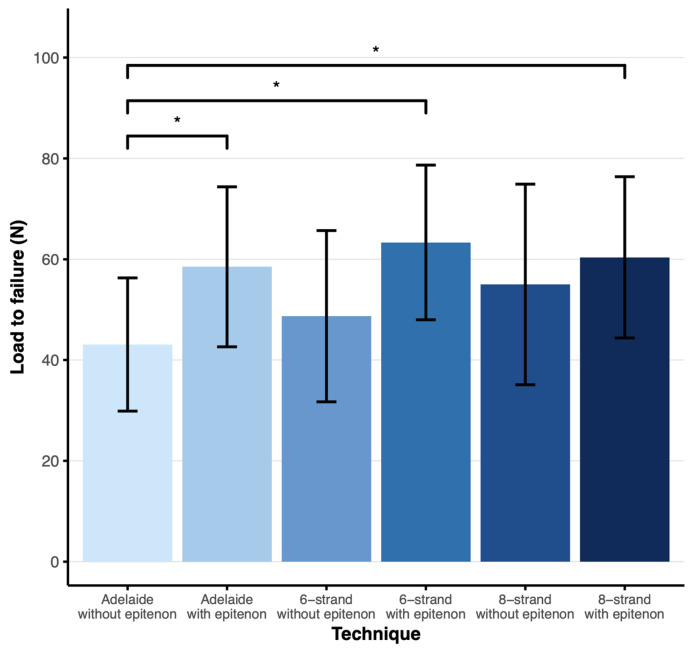
Load to failure plot showing the mean load to failure in N with error bars representing the SD. * Significant difference *p* < 0.05 as found by Tukey’s HSD test for multiple comparisons. N = 19 (6-strand without epitenon), n = 20 (Adelaide ± epitenon, 6-strand with epitenon, 8-strand ± epitenon).

The mean load to failure in the Adelaide without epitenon group was 43 N (SD = 13), compared to 58 N (SD = 16) in the Adelaide with epitenon group, 49 N (SD = 17) in the six-strand without epitenon group, 63 N (SD = 15) in the six-strand with epitenon group, 55 N (SD = 20) in the eight-strand without epitenon group, and 60 N (SD = 16) in the eight-strand with epitenon group (Figure 2).

The mean load to failure of the Adelaide with epitenon group, six-strand with epitenon group, and eight-strand with epitenon group were significantly higher than the mean load to failure of the Adelaide without epitenon group (*p* < 0.05, 95% CI 0.44–30.41, *p* < 0.05, 95% CI 5.28–35.24, and *p* < 0.05, 95% CI 2.32–32.28, respectively). There were no significant differences between other groups.

### 3.2. Failure Cause

In all groups, except the six-strand without epitenon group, suture breakage was the primary cause of failure, ranging from 55% to 75% of cases. In the remaining cases, failure occurred due to suture pullout from the tendon. In the six-strand without epitenon group, suture pullout was the primary cause of failure, observed in 58% of cases. No instances of tendon slippage from the testing clamps were observed in any group (Table 3). No association was found between suture technique and cause of failure (Χ2(2) ≥ 6.16, *p* > 0.05).

### 3.3. CSA Data

**Figure 3:**
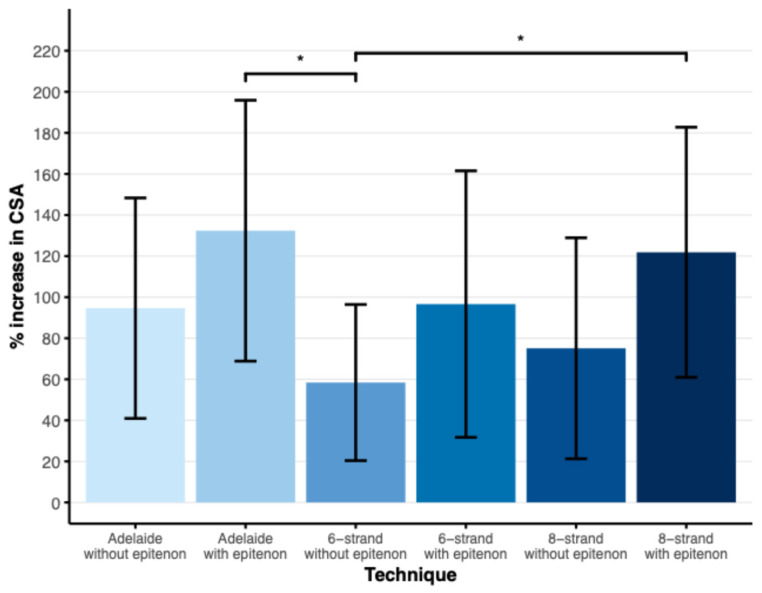
Plot showing the mean percentage increase in CSA of the tendon after suturing, error bars represent the SD. * Significant difference *p* < 0.05 as found by Tukey’s HSD test for multiple comparisons. N = 16 (Adelaide ± epitenon, 8-strand without epitenon), n = 19 (6-strand without epitenon), n = 20 (6-strand with epitenon, 8-strand with epitenon).

The mean increase in CSA after suturing was 95% in the Adelaide without epitenon group, 132% in the Adelaide with epitenon group, 58% in the six-strand without epitenon group, 97% in the six-strand with epitenon group, 75% in the eight-strand without epitenon group, and 122% in the eight-strand with epitenon group (Figure 3). The Adelaide with epitenon and eight-strand with epitenon groups showed a significantly greater increase in CSA compared to the six-strand without epitenon group (*p* < 0.05).

## 4. Discussion

This study aimed to assess whether six- or eight-strand looped core suture techniques provide greater tensile strength without increasing repair bulkiness compared to the conventional four-strand Adelaide repair. We found that the eight-strand without epitenon suture achieved a similar load to failure as the Adelaide with epitenon, while also resulting in a smaller increase in CSA. In addition, the six-strand with epitenon demonstrated a comparable load to failure to both the Adelaide with epitenon and the eight-strand with epitenon, suggesting that it is a mechanically adequate alternative to the eight-strand repair, potentially requiring less tissue manipulation. These results suggest that eight core strands may provide sufficient tensile strength to allow omission of the epitenon suture under laboratory conditions. This leads to less repair bulkiness and possibly higher surgical efficiency, although surgical time was not measured. In this context, the availability of multiple mechanically adequate repair techniques supports a more individualized approach to tendon repair, allowing surgeons to tailor technique selection based on clinical scenario rather than relying on a single standardized method.

Our findings are consistent with previous studies, demonstrating that increasing the number of core strands crossing the repair site enhances the tensile strength of the repair [9,12,13,14]. This is particularly relevant in the context of early active rehabilitation, as the repair is subjected to greater forces while the tendon is still healing [12,14,15,16]. Tendon forces of up to 38 N have been reported during early active rehabilitation [17], and a minimum load-to-failure threshold of 50 N is commonly used in laboratory studies to provide an adequate safety margin [12]. In the present study, the Adelaide repair with epitenon suture, the six-strand repair with epitenon suture, and both eight-strand repairs exceeded this threshold. In contrast, the Adelaide repair without epitenon suture and the six-strand repair without epitenon suture did not, with mean loads to failure of 43 N and 49 N, respectively. Among the techniques that met the 50 N threshold, the eight-strand repair without epitenon suture may represent a simplified alternative in terms of tensile strength, as it avoids epitenon suturing while maintaining an adequate load to failure.

While previous literature has shown that adding epitenon sutures in repairs with up to six core strands improves load to failure, reduces gapping, and decreases gliding resistance [18,19,20,21], more recent literature shows that epitenon sutures may not be required in six-strand repairs [22,23]. Notably, our findings show that adding an epitenon suture in the six- and eight-strand repair group did not result in a significant increase in load to failure. This suggests that the strength benefits of an epitenon suture might be less impactful in repairs with a higher number of core strands, as they already provide sufficient tensile strength. Additionally, repairs with a higher number of core sutures have been shown to have a higher resistance to gapping, which could reduce the need for epitenon sutures [13,14]. However, due to the conflicting results in the existing literature, more research is needed to confirm our findings.

The existing literature also provides conflicting evidence regarding the relationship between repair bulk and gliding resistance [9,24]. In the present study, gliding resistance was not assessed. However, the eight-strand repair without an epitenon suture showed a significantly smaller increase in CSA compared with the conventional Adelaide repair with an epitenon suture, indicating a reduction in repair bulk. Whether this translates into improved functional gliding remains to be determined in future studies.

In the present study, failure of the tendon repairs occurred primarily through suture rupture or pull-out from the tendon. Importantly, five of the six repair techniques demonstrated mean load-to-failure values exceeding the laboratory threshold generally considered sufficient for early active rehabilitation [12]. This suggests that these techniques provide adequate initial biomechanical safety. The observed failure causes nonetheless underscore the importance of both the mechanical properties of the suture material and the integrity of the knot configuration. Suture characteristics such as tensile strength, elasticity, and handling may influence the likelihood of rupture, whereas insufficient knot security or suboptimal suture placement within the tendon can predispose repairs to pull-out. In clinical practice, careful selection of suture material, adherence to established knot-tying techniques, and meticulous placement of core and epitenon sutures are therefore critical to minimizing failure risk.

From a personalized medicine standpoint, these findings suggest that flexor tendon repair strategies may be adapted to specific patient and injury characteristics. For example, an eight-strand repair without epitenon suture may be advantageous in patients requiring early active rehabilitation or in settings where minimizing repair bulk is critical, while a six-strand repair with epitenon suture may offer sufficient strength with reduced tissue manipulation in less demanding clinical scenarios. Such flexibility may be particularly relevant in patients with compromised tendon quality, complex injuries, or anticipated challenges with postoperative compliance. Rather than identifying a universally optimal technique, this study supports a stratified approach to repair selection based on biomechanical requirements and clinical context.

This study has several limitations. First, a cadaveric tendon model was used, precluding the assessment of biological healing processes such as vascularity, inflammation, and remodeling that influence tendon repair strength in vivo [16]. Second, biomechanical testing was limited to monotonic load-to-failure measurements. In clinical practice, repaired tendons are subjected to cyclic loading during rehabilitation. Although previous studies suggest that higher-strand repairs perform better under cyclic loading [12,25,26], this was not directly evaluated in the present study. Third, gapping resistance is an important factor in tendon healing [27,28], which was not specifically measured. Consequently, conclusions regarding this parameter—particularly in relation to omission of the epitenon suture—are limited. Both cyclic loading and gapping analysis would strengthen the causal interpretation of the results. Broad methodological caution remains imperative when interpreting biomechanical results and drawing conclusions on clinical causality [29]. In addition, while a larger sample size may be required to detect smaller effect sizes, the present study was designed to assess biomechanical equivalence rather than superiority. Within this context, the sample size was considered sufficient to support conclusions regarding comparable load-to-failure performance between techniques. Finally, the core suture material used in the four-strand Adelaide differed from the core suture material used in the six- and eight-strand techniques. Potential biomechanical differences between these sutures should be considered when interpreting the results.

## 5. Conclusions

This study suggests that an eight-strand looped core suture technique without epitenon suture may represent a biomechanically sound alternative to the conventional four-strand Adelaide repair with epitenon suture, achieving a comparable load to failure. By eliminating the epitenon suture, the eight-strand looped approach minimizes repair bulk and simplifies the repair while maintaining adequate tensile strength. In addition, the six-strand with epitenon demonstrated similar tensile strength to both the Adelaide with epitenon and the eight-strand with epitenon, indicating that it may be a mechanically adequate alternative to higher-strand repairs, with less tissue manipulation. These findings support a more individualized approach to flexor tendon repair, in which the choice of repair technique can be tailored to biomechanical demands and clinical context rather than applying a single uniform technique. The next step should be to evaluate high-strand repairs without epitenon suture under cyclic loading conditions, including assessment of gapping resistance and reconstruction time. In addition, in vivo studies are required to determine the effect of increased core suture material on tendon healing with a view to personalizing medical treatment.

## Figures and Tables

**Table 1:** Tendons in each group with complete load to failure data.

Suture Technique	FDP 2	FDP 3	FDP 4	FDP 5	Total Tendons
Adelaide without epitenon	5	5	5	5	20
Adelaide with epitenon	5	5	5	5	20
6-strand without epitenon	4	5	5	5	19
6-strand with epitenon	5	5	5	5	20
8-strand without epitenon	5	5	5	5	20
8-strand with epitenon	5	5	5	5	20

Table showing the distribution of tendons within each group, along with the total number of tendons per group for the load to failure and failure cause analyses.

**Table 2:** Tendons in each group with complete CSA data.

Suture Technique	FDP 2	FDP 3	FDP 4	FDP 5	Total Tendons
Adelaide without epitenon	4	4	4	4	16
Adelaide with epitenon	4	4	4	4	16
6-strand without epitenon	4	5	5	5	19
6-strand with epitenon	5	5	5	5	20
8-strand without epitenon	4	4	4	4	16
8-strand with epitenon	5	5	5	5	20

Table showing the distribution of tendons within each group, along with the total number of tendons per group for the CSA analysis.

**Table 3:** Failure cause of suture repair.

Suture Technique	Failure Cause	
	Suture Pullout from Tendon (n; %)	Suture Breakage (n; %)
Adelaide without epitenon	6; 30%	14; 70%
Adelaide with epitenon	9; 45%	11; 55%
6-strand without epitenon	11; 58%	8; 42%
6-strand with epitenon	5; 25%	15; 75%
8-strand without epitenon	8; 40%	12; 60%
8-strand with epitenon	10; 50%	10; 50%

Table showing the distribution of tendons (n; %) by failure cause within each group. N = 19 (6-strand without epitenon), n = 20 (Adelaide ± epitenon, 6-strand with epitenon, 8-strand ± epitenon).

## Data Availability

The raw data supporting the conclusions of this article will be made available by the authors on request.

## Abbreviations

The following abbreviations are used in this manuscript:

FDP	Flexor Digitorum Profundus
N	Newton
CSA	Cross-Sectional Area
SD	Standard Deviation
CI	Confidence Interval
